# Effect of Soy Protein Supplementation on Muscle Adaptations, Metabolic and Antioxidant Status, Hormonal Response, and Exercise Performance of Active Individuals and Athletes: A Systematic Review of Randomised Controlled Trials

**DOI:** 10.1007/s40279-023-01899-w

**Published:** 2023-08-21

**Authors:** Reza Zare, Asli Devrim-Lanpir, Silvia Guazzotti, Ali Ali Redha, Konstantinos Prokopidis, Daniele Spadaccini, Roberto Cannataro, Erika Cione, Menno Henselmans, Alan A. Aragon

**Affiliations:** 1 Meshkat Sports Complex, Karaj, Alborz Province Iran; 2 Arses Sports Complex, Karaj, Alborz Province Iran; 3 Department of Nutrition and Dietetics, Faculty of Health Sciences, Istanbul Medeniyet University, Istanbul, Turkey; 4 School of Health and Human Performance, Dublin City University, Dublin 9, D09 V209 Ireland; 5 Department of Translational Medicine (DiMeT), Center for Translational Research on Autoimmune and Allergic Diseases-CAAD, University of Piemonte Orientale, 28100 Novara, Italy; 6 The Department of Public Health and Sport Sciences, University of Exeter Medical School, Faculty of Health and Life Sciences, University of Exeter, Exeter, EX1 2LU UK; 7 Centre for Nutrition and Food Sciences, Queensland Alliance for Agriculture and Food Innovation (QAAFI), The University of Queensland, Brisbane, QLD 4072 Australia; 8 Department of Musculoskeletal Biology, Institute of Life Course and Medical Sciences, University of Liverpool, Liverpool, L7 8TX UK; 9 Society of Meta-Research and Biomedical Innovation, London, UK; 10 Department of Health Sciences, University of Piemonte Orientale, 28100 Novara, Italy; 11 Department of Pharmacy, Health and Nutritional Sciences, University of Calabria, 87036 Rende, Italy; 12 GalaScreen Laboratory, Department of Pharmacy, Health and Nutritional Sciences, University of Calabria, 87036 Rende, Italy; 13 The International Scientific Research Foundation for Fitness and Nutrition, David Blesstraat 28HS, 1073 LC Amsterdam, The Netherlands; 14 Department of Family and Consumer Sciences, California State University, Northridge, CA USA

## Abstract

**Background:**

Protein supplements are important to maintain optimum health and physical performance, particularly in athletes and active individuals to repair and rebuild their skeletal muscles and connective tissues. Soy protein (SP) has gained popularity in recent years as an alternative to animal proteins.

**Objectives:**

This systematic review evaluates the evidence from randomised controlled clinical trials of the effects of SP supplementation in active individuals and athletes in terms of muscle adaptations, metabolic and antioxidant status, hormonal response and exercise performance. It also explores the differences in SP supplementation effects in comparison to whey protein.

**Methods:**

A systematic search was conducted in PubMed, Embase and Web of Science, as well as a manual search in Google Scholar and EBSCO, on 27 June 2023. Randomised controlled trials that evaluated the applications of SPs supplementation on sports and athletic-related outcomes that are linked with exercise performance, adaptations and biomarkers in athletes and physically active adolescents and young adults (14 to 39 years old) were included, otherwise, studies were excluded. The risk of bias was assessed according to Cochrane’s revised risk of bias tool.

**Results:**

A total of 19 eligible original research articles were included that investigated the effect of SP supplementation on muscle adaptations (*n* = 9), metabolic and antioxidant status (*n* = 6), hormonal response (*n* = 6) and exercise performance (*n* = 6). Some studies investigated more than one effect. SP was found to provide identical increases in lean mass compared to whey in some studies. SP consumption promoted the reduction of exercise-induced metabolic/blood circulating biomarkers such as triglycerides, uric acid and lactate. Better antioxidant capacity against oxidative stress has been seen with respect to whey protein in long-term studies. Some studies reported testosterone and cortisol fluctuations related to SP; however, more research is required. All studies on SP and endurance performance suggested the potential beneficial effects of SP supplementation (10–53.3 g) on exercise performance by improving high-intensity and high-speed running performance, enhancing maximal cardiac output, delaying fatigue and improving isometric muscle strength, improving endurance in recreational cyclists, increasing running velocity and decreasing accumulated lactate levels; however, studies determining the efficacy of soy protein on VO_2_max provided conflicted results.

**Conclusion:**

It is possible to recommend SP to athletes and active individuals in place of conventional protein supplements by assessing their dosage and effectiveness in relation to different types of training. SP may enhance lean mass compared with other protein sources, enhance the antioxidant status, and reduce oxidative stress. SP supplementation had an inconsistent effect on testosterone and cortisol levels. SP supplementation may be beneficial, especially after muscle damage, high-intensity/high-speed or repeated bouts of strenuous exercise.

**Supplementary Information:**

The online version contains supplementary material available at 10.1007/s40279-023-01899-w.

## Key Points

**Table:** 

Soy protein (SP) is a sustainable and plant-sourced protein that is rich in nutrients (e.g., isoflavones) that could be absent in animal-sourced proteins. Yet, the essential amino acid content of SP is lower than some animal-sourced proteins such as whey protein. Thus, a holistic critical evaluation of the effectiveness of SP supplementation is necessary.
SP supplementation may be an effective alternative to whey in promoting optimal muscle mass and strength gains, at least in young populations, utilizing a protein intake of ≥ 1.6 g/kg/day. However, this was based on a limited number of trials.
SP supplementation has shown promising antioxidant effects in comparison with whey protein supplementation due to its rich nutritional composition that can aid in oxidative stress.
The influence of SP supplementation on anabolic hormones is not clear and requires further investigation.
SP supplements can be beneficial to enhance exercise performance, especially those that are associated with muscle damage or strenuous exercise.

## Introduction

Intake of dietary protein is a crucial part of modern nutritional methods, especially to optimise post-exercise recovery [1]. The most common protein sources used in sports supplementation are whey (a by-product of cheese manufacturing), casein from milk, ovalbumin from egg whites, legumes (mainly soy and peas) and cereal proteins (such as rice) [2]. Proteins can support the human body to build muscle tissue quicker and more efficiently. Consuming protein-rich supplements as a pre- and/or post-workout supplement can significantly increase muscle protein synthesis [3].

At a molecular level, protein intake, as well as mechanical loading, stimulates increased rates of mixed skeletal muscle protein synthesis via the mechanistic target of rapamycin complex 1 (mTORC1) signalling cascade, a master regulator of protein synthesis that translocates toward the cell periphery [4]. The peripherical localization of mTORC1 is strategic in human skeletal muscle fibres because of close proximity to focal adhesion complexes and in relation with upstream activators (e.g., Akt), downstream targets [i.e., ribosomal protein S6 kinase beta-1, p70S6K and eukaryotic translation initiation factor 4E (eIF4E)-binding protein 1, 4E-BP1], and microvasculature, where L-type amino acid may enter into the fibres [5]. Recent research evidence that the phosphorylation state of mTOR^Ser2448^ and p70S6K^Thr389^ may be further enhanced by protein consumption during the first 2 h of post-exercise recovery [6].

It is well known that low to moderate level of oxidative stress may provide advantages by improving endogenous antioxidant defences [7]. However, high-intensity or prolonged exercise may increase exercise-induced oxidative stress, defined as the imbalance between reactive oxygen species and antioxidant defence in favour of oxidants that cause an increase in reactive oxygen species (ROS) in the body, thus impairing exercise performance by causing oxidative damage to skeletal muscle fibres or by causing muscle fatigue [8]. According to some research, the potential impact of soy protein on antioxidant system is attributed to its bioactive antioxidants, including isoflavones [9]. It is thought that isoflavones exert their beneficial effects as an antioxidant directly by quenching free oxygen species, especially with genistein and daidzein isoflavones, or indirectly by increasing antioxidant scavenging enzymes [10]. A systematic review and meta-analysis of 24 randomised controlled trials on soy isoflavones and oxidative stress biomarkers revealed that soy protein significantly decreased malondialdehyde, a well-known biomarker of oxidative stress, and increased antioxidant biomarkers, including total antioxidant capacity, superoxide dismutase activity and total reactive antioxidant potential compared to the control group [11]. Another systematic review and meta-analysis of 51 RCTs on soy supplementation and inflammatory biomarkers reported a significant decrease in plasma C-reactive protein (CRP) concentrations after soy, although plasma TNF-α and IL-6 concentrations were unchanged [12]. One point to consider is that these studies included in these meta-analyses were conducted on either various patients or healthy sedentary individuals. Although soy protein is a promising macronutrient that may improve exercise performance by reducing exercise-induced oxidative stress, to our knowledge, there are no systematic reviews evaluating the effect of soy protein on active individuals and athletes. It is important to critically review the studies which have investigated the effect of soy protein supplementations on athletes and active individuals systematically to suggest its overall influence on antioxidant and inflammatory biomarkers. That is because the effect of soy protein may vary depending on training regimen (e.g., exercise type), duration of intervention, individual’s training/exercise experience, and the effect of timing and dose of supplementation.

In recent years, the environmental effect of nutritional supplement production has received a great deal of attention, and there has been a lot of interest in producing proteins from plant-sources rather than those from animals [13]. Other common reasons that people consume vegetarian products include religious traditions, ethical considerations and health benefits, such as providing a better plasma lipid profile and reducing high arterial blood pressure, thus reducing the risk of cardiometabolic disease [14]. Considering the high use of plant-based protein sources in athletes in recent years [14, 15], several studies have investigated the composition and efficacy of the most common plant-based proteins, including soy, wheat, pea, rice and potato protein [16–20]. Plant-based proteins can include all of the essential amino acids (EAAs); however, they are less abundant compared with animal sources, and the presence of anti-nutrients such as oxalate, phytate, tannins, lectins and trypsin inhibitors that inhibit proteolysis limits their digestion and absorption [21]. Protein digestibility reveals the fraction of ingested amino acids that can be utilised by the body. Typically, animal proteins are highly digestible (> 90%), rendering them available to be absorbed and metabolized [22]. Compared with general low-digestibility plant proteins (75–80%), soy protein (SP) may provide advantages with its higher digestibility (95%) [23] and better composition of EAAs. However, although SPs are considered high-quality protein, they are low in leucine (6%) and sulphur-containing amino acids, such as methionine and cysteine compared with animal-based proteins [24]. Studies comparing the effectiveness of SP with whey protein have shown equivocal results [25–27]. Although previous research found anabolic superiority of whey protein supplementation [28, 29], a meta-analysis of nine long-term studies investigating the efficacy of equal amounts of supplemental soy and animal proteins (a majority from dairy) on muscle mass and muscle strength in response to resistance exercise found no significant difference in lean body mass and strength [30]. However, studies included in the meta-analysis were mostly (six out of nine) conducted on individuals that did not engage in resistance exercise for more than a year. Furthermore, of the nine studies in the meta-analysis, three favoured animal protein, and six studies showed no significant advantage of either protein type. In none of the studies was soy the superior performer.

The ergogenic effect of protein supplementation is well known in sports nutrition; however, the effect of SPs on exercise and sports-related outcomes in active and athletic population has not been comprehensively evaluated. This systematic review aims to discuss the effectiveness of SP supplementation critically and comprehensively on muscle adaptations, metabolic and antioxidant status, hormonal response and exercise performance of active individuals and athletes based on randomised controlled trials. In addition, where applicable, the potential differences between SP supplementation and whey protein supplementation as an animal-sourced protein supplement were discussed.

## Methods

The protocol of this systematic review is based on the recommendation of the Preferred Reporting Items for Systematic Reviews and Meta-Analyses (PRISMA) statement [31]. The protocol of this systematic review was registered at the Open Science Framework (https://doi.org/10.17605/OSF.IO/JY8VA).

### Literature Search

A systematic search was conducted in three electronic databases (PubMed, Embase and Web of Science) for relevant studies on 16 September 2022 and was reconducted on 27 June 2023. The search strategy was based on including (“soy protein” OR “soya protein”) AND (“sport” OR “exercise” OR “fitness” OR “bodybuilding” OR “athlete” OR “training”). Manual search was also performed in Google Scholar and EBSCO. The records obtained from the different electronic databases were imported into Microsoft Excel and duplicates were removed. Two reviewers (R.Z. and A.AR.) were responsible for independently screening each article’s title, abstract and full text. A third reviewer (S.G.) arbitrated when needed.

### Study Selection

The inclusion criteria of this systematic review were: (i) human study, (ii) randomised controlled clinical trial, (iii) participants supplemented with SP, SP-based products (SP being the major component) or soy peptides, (iv) participants who were physically active or recreationally active individuals [performing at least 1 h of exercise per day for at least 3 days a week (minimum requirement)], regularly trained or athletes (this included athletes who performed a type of sports activity as a part of a sports team or as independent professional/semi-professional/recreational athletes), (v) participants in the age range of 14–17 years (adolescents) and 18–39 years (young adults), (vi) study investigated sports and athletic-related outcomes that were linked with exercise performance, adaptations and biomarkers (including muscle synthesis, muscle growth, muscle strength, metabolic status/blood circulating biomarkers, redox status, and hormonal response), and (vii) studies were peer-reviewed and written in English. The search strategy excluded (i) non-clinical studies, (ii) untrained, physically inactive (do not perform at least 1 h of exercise per day for at least 3 days a week), sedentary (energy expenditure of 1.5 metabolic equivalent task (MET) or less) or unhealthy participants, (iii) participants over 40 years old [this is because above this age the individual starts to experience changes in muscle mass and strength, muscle fibre composition, hormonal changes, and variations in training response, exercise capacity, recovery and adaptation rate], (iv) studies evaluating outcomes not relevant to exercise performance and athletic status, (v) non-English studies, and (vi) comments, editorials or reviews. The obtained records were screened, and studies were selected based on the beforementioned criteria. To decide which studies were eligible for each synthesis, the study intervention characteristics were listed in a table and comparing against the planned groups for each synthesis.

### Data Extraction

Two reviewers (R.Z. and A.AR.) extracted the data from the eligible studies. The characteristics of the included studies are summarized in Tables 1, 2, 3 and 4. The following information was extracted: study design, characteristics of participants (number, sports performed, sex and age), supplementation intervention (number of participants, supplementation form and supplementation dosage), placebo/comparable intervention (number of participants, form and dosage), study duration, experimental design and main outcomes. The data were divided on the basis of outcomes in four different tables: muscle adaptations (Table 1), metabolic and antioxidant status (Table 2), hormonal response (Table 3) and exercise performance (Table 4). A meta-analysis was not conducted due to the heterogeneity in study designs and variations in the biomarkers selected for the different outcomes.

**Table 1:** Summary of randomised controlled trials evaluating the effect of soy-based supplementation on muscle adaptations of active individuals and athletes

Author, year [reference]	Type of study	Participants	Soy protein-supplemented group	Placebo/comparison group(s)
**Acute supplementation design studies**				
Wilkinson et al., 2007 [43]	Randomised single-blind crossover trial	Healthy young active males engaged in resistance training: age average 21.6 ± 0.3 years old	*n* = 8 Received 500 mL/day of soy beverage (745 kJ, 18.2 g protein, 1.5 g fat and 23 g carbohydrate as maltodextrin)	*n* = 8 Received 500 mL/day of fluid non-fat milk (745 kJ, 18.2 g protein, 1.5 g fat and 23 g carbohydrate as lactose)
Churchward-Venne et al., 2019 [17]	Randomised double-blind parallel-group trial	Healthy young recreationally active men: age average 23 ± 0.4 years old	*n* = 12 Received 590 mL of soy beverage or soy supplemented with free leucine (20 g of soy protein and 45 g of carbohydrate). The amount of leucine was equivalent to that present in whey protein	*n* = 12 Received 590 mL of whey beverage (20 g of whey protein and 45 g of carbohydrate)

Study duration	Experimental design	Main outcomes
1 day per study condition	Participants consumed fluid milk or a soy protein beverage after a bout of resistance exercise, and the arterial–venous amino acid balance and muscle fractional synthesis rates were measured Washout period ≥ 1 week	Both supplements had a positive effect on net protein balance. Consumption of milk resulted in a higher net balance (*p* < 0.05), and the fractional synthesis rate in muscle (0.10 ± 0.01%/h) compared with soy consumption (0.07 ± 0.01%/h; *p* = 0.05)
1 day per study condition	Participants consumed one of the beverages after concurrent resistance- and endurance-type exercise. Blood and muscle biopsy samples were collected from participants over a 360 min post-exercise recovery period to examine postprandial myofibrillar (MyoPS) and mitochondrial (MitoPS) protein synthesis rates, and the associated signalling through the mammalian target of rapamycin complex 1 (mTORC1)	Participants who consumed whey protein and soy protein + leucine had significantly higher postprandial peak plasma leucine concentrations (322 ± 10 and 328 ± 14 μmol/L, respectively) compared with soy protein (216 ± 6 μmol/L) (*p* < 0.05). The synthesis rates of MyoPS and MitoPS over the entire 360 min recovery period did not differ between treatments (*p* > 0.05). In addition, signalling through mTORC1^Ser2448^, p70S6k^Thr389^, 4E-BP1^Thr37/46^ and rpS6^Ser235/236^ was also similar between the different treatments

Author, year [reference]	Type of study	Participants	Soy protein-supplemented group	Placebo/comparison group(s)
Reidy et al., 2014 [42]	Randomised double-blind trial	Healthy young recreationally active men: age average 23 ± 1.0 years old	*n* = 8 Received 300 mL of soy–dairy protein blend beverage (20.1 ± 0.9 g total protein (providing 1.9 ± 0.1 g leucine, 1.0 ± 0.1 g phenylalanine, 1.3 ± 0.02 g valine, and 9.0 ± 0.4 g EAA) composed of 50% protein from sodium caseinate, 25% protein from whey protein isolate, and 25% protein from soy protein isolate)	*n* = 8 Received 300 mL of whey protein beverage (17.3 ± 0.9 g of protein (providing 1.9 ± 0.1 g leucine, 0.6 ± 0.1 g phenylalanine, 1.1 ± 0.1 g valine, and 8.7 ± 0.5 g EAA) composed of 100% whey protein isolate)
Reidy et al., 2013 [45]	Randomised double-blind controlled trial	Healthy young recreationally active men: age range 18–30 years	*n* = 10 Received 0.35 g total protein /kg lean mass (~ 18 g) of soy protein-based blend beverage (composed of 25% protein from soy protein isolate, 25% protein from whey protein isolate, and 50% protein from sodium caseinate) beverage an hour after exercise	*n* = 9 Received 0.30 g total protein /kg lean mass (~ 19 g) of whey protein isolate beverage an hour after exercise

Study duration	Experimental design	Main outcomes
1 day per study condition	Participants consumed one of the beverages an hour after a bout of high-intensity leg resistance exercise. Phenylalanine net balance and transport rate into the skeletal muscle of participants were measured using stable isotopic methods in combination with femoral arteriovenous blood sampling and muscle biopsies obtained at rest and 3 and 5 h postexercise	A significant increase (*p* < 0.05) in the levels of phenylalanine transport into muscle and mRNA expression of selected amino acid transporters [system L amino acid transporter 1/solute-linked carrier (SLC) 7A5, CD98/SLC3A2, system A amino acid transporter 2/SLC38A2, proton-assisted amino acid transporter 1/SLC36A1, cationic amino acid transporter 1/SLC7A1] was determined in both groups. Yet, participants who received the soy–dairy protein blend experienced a significantly long and positive net phenylalanine balance (*p* < 0.05) during postexercise recovery in comparison to those who received whey protein
1 day per study condition	Participants received the assigned supplement after an hour of completing high-intensity leg resistance exercises. The mixed-muscle protein fractional synthetic rate was measured using stable isotopic methods and the mammalian target of rapamycin (mTORC1) signalling was assessed using western blotting	Soy protein-based blend supplementation caused a lower initial rise in blood branched-chain amino acid, in comparison with whey protein supplementation; however, it maintained high levels of blood amino acids later into recovery (*p* < 0.05). The mixed-muscle protein fractional synthetic rate increased in both groups during post-exercise; however, it remained significantly elevated in the soy group (soy group, 0.087 ± 0.003%, whey group, 0.074 ± 0.010%) (*p* < 0.05). The increase in mTORC1 signaling was similar in both groups, however, no increase was observed in S6K1 phosphorylation in the whey group at 5 h post-exercise (*p* < 0.05)

Author, year [reference]	Type of study	Participants	Soy protein-supplemented group	Placebo/comparison group(s)
**Chronic supplementation design studies**				
Lynch et al., 2020 [41]	Randomised double-blind parallel-group trial	Healthy young recreationally active individuals: age 18–35 years	*n* = 22 Received 26 g of soy protein isolate (containing 2 g of leucine)/day as a beverage	*n* = 26 Received 19 g of whey protein isolate (containing 2 g of leucine)/day as a beverage
Hartman et al., 2007 [40]	Randomise control parallel 3-group longitudinal trial	Healthy young recreationally active weightlifter men: age range 18–30 years	*n* = 19 Received 500 mL of fat-free soy protein drink immediately and 1 h after exercise	*n* = 19 Received 500 mL of fat-free milk immediately and 1 h after exercise (Placebo group: *n* = 19 Received 500 mL of flavoured fluid containing carbohydrate that was isoenergetic with the other drinks)

Study duration	Experimental design	Main outcomes
12 weeks	Participants consumed the supplement on a daily basis and engaged in supervised resistance training 3 times/week (post-workout on training days). Muscle strength and growth were measured prior to and after 6 and 12 weeks of training. Changes in lean body mass, peak torque, muscle thickness, adiposity, and total body mass were measured	A significant increase in total body mass (0.68 kg; 95% CI: 0.08, 1.29 kg; *p* < 0.001), lean body mass (1.54 kg; 95% CI: 0.94, 2.15 kg; *p* < 0.001), and peak torque of leg extensors (40.27 Nm; 95% CI: 28.98, 51.57 Nm, *p* < 0.001) and flexors (20.44 Nm; 95% CI: 12.10, 28.79 Nm; *p* < 0.001) was observed in the whey protein isolate and soy protein isolate-supplemented groups. No significant difference was observed in changes in vastus lateralis muscle thickness in both groups (0.12 cm; 95% CI: − 0.01, 0.26 cm; *p* = 0.08). Overall, no significant differences were observed between the groups (*p* > 0.05)
12 weeks	Participants performed 5 days/week of resistance exercise training on a rotating whole-body split routine. They consumed the assigned drink immediately and 1 h after exercise. The muscle fibre size, maximal strength and body composition by dual-energy X-ray absorptiometry were measured before and after training	An increase in type II muscle fibre area was observed in all the groups; however, the increase in the milk group was significantly higher (*p* < 0.05). Type I muscle fibre area increased in the soy and milk groups, but the increase in the milk group was significantly higher than the control group (*p* < 0.05). A difference in the strength between-group was not observed. The fat- and bone-free mass increased in all groups, with a significant and greater increase in the milk group compared with other groups (*p* < 0.05)

Author, year [reference]	Type of study	Participants	Soy protein-supplemented group	Placebo/comparison group(s)
Reidy et al., 2016 [44]	Randomised double-blind placebo-controlled trial	Healthy young recreationally active men: age range 18–30 years	*n* = 23 Received 22 g/day of soy protein-based blend beverage (comprised of 25% protein from soy protein isolate, 25% protein from whey protein isolate, and 50% protein from sodium caseinate)	*n* = 22 Received 22 g/day of whey protein isolate beverage (Placebo group: *n* = 23 Received 22 g/day of isocaloric maltodextrin beverage)
Reidy et al., 2017 [46]	Randomised double-blind placebo-controlled trial	Healthy young recreationally active men: age range 18–30 years	*n* = 22 Received 22 g/day of soy protein-based blend beverage (comprised of 25% protein from soy protein isolate, 25% protein from whey protein isolate, and 50% protein from sodium caseinate)	*n* = 15 Received 22 g/day of whey protein isolate beverage (Placebo group: *n* = 17 Received 22 g/day of isocaloric maltodextrin beverage)
Brown et al., 2004 [39]	Randomised double-blind placebo-controlled trial	Experienced weightlifters with at least 1 year or more experience in strength training: age range 19–25 years	*n* = 9 Received micronutrient-fortified soy protein bars (33 g protein/day)	*n* = 9 Received micronutrient-fortified whey protein bars (33 g protein/day) (Control group: *n* = 9 Performed training but did not receive any nutritional bars)

Study duration	Experimental design	Main outcomes
12 weeks	Participants performed 3 days/week of resistance exercise training. They consumed the assigned supplement after exercise (during exercise days) Their muscle strength, thigh muscle thickness, myofiber cross-sectional area and lean body mass were measured before and after 6 and 12 weeks of resistance exercise training	The lean body mass of all participants increased significantly (*p* < 0.01), with a greater change in the soy protein-based blend group in comparison with the whey group (0.92 kg; 95% CI: 20.12, 1.95 kg; *p* = 0.09). The muscle strength, thigh muscle thickness, and myofiber cross-sectional area of all participants increased significantly (*p* < 0.05); however, no significant differences between the groups were observed (*p* > 0.10)
12 weeks	Participants were involved in supervised whole-body progressive resistance training for 3 days/week and consumed the assigned supplement after exercise (during exercise days). Their lean mass, vastus lateralis myofibre type-specific cross-sectional area, satellite cell content and myonuclear addition were assessed before and after resistance training	Both protein-supplemented groups experienced a significantly higher greater whole-body lean mass percentage change (*p* ≤ 0.05). All groups showed similar leg muscle hypertrophy and vastus lateralis myofibre type-specific cross-sectional area. The elevation in myosin heavy chain I and II myofibre satellite cell content and myonuclei content were also similar in all groups
9 weeks	Intervention groups consumed protein bars and had a short-term power-based weight training program. Pre- and post-intervention lean body mass were analysed by hydrostatic weighing	Both protein bars showed a significant increase (*p* < 0.05) in lean body mass in conjunction with a short-term power-based weight training program

*CI* confidence interval

**Table 2:** Summary of randomised controlled trials evaluating the effect of soy-based chronic supplementation on the metabolic and antioxidant status of active individuals and athletes

Author,year [reference]	Type of study	Participants	Soy protein-supplemented group
Berg et al., 2012 [47]	Randomised controlled trial	Clinically healthy sports student: age average 23.6 ± 1.9 years	*n* = 15* Received two 50 g/day servings of soy protein-based supplement (Almased® containing 53.3 g protein, 30.5 g carbohydrates, 2.0 g fat, 354 kcal per 100 g)
Hill et al., 2004 [48]	Randomised double-blind trial	Recreationally trained young adult males: age range 18–25 years old	*n* = 9 Received 39 g/day of SUPRO® isolated soy protein supplement as a beverage
Wenxue, 2013 [51]	Randomised placebo-controlled trial	Second grade male volleyball players: average age 20.2 ± 1.9 years	*n* = 6 Received soy protein hydrolysate-based beverage (10 g of soy peptide and 30 g of sugar) as a post-training drink every training day

Placebo/comparison group(s)	Study duration	Experimental design	Main outcomes
*n* = 15* Control group (no supplementation)	6 weeks	Participants performed moderate endurance training (60 min per day, 5 times a week) at the aerobic threshold. Pre- and post-intervention metabolic status (glucose, lactate, triglycerides, urea, uric acid, ammonia) and inflammation markers (creatine kinase, lactate dehydrogenase, myoglobin, high-sensitivity C-reactive protein, interleukin-6, and interleukin-10) were measured	The supplemented group showed lower lactate values following the intervention. In addition, they showed significantly lower differences in the exercise-induced increase of metabolic parameters (triglycerides and uric acid) in the post-exercise recovery period (*p* < 0.05)
*n* = 9 Received 39 g/day of whey protein supplement as a beverage	4 weeks	Participants received the supplement before a session of moderate intensity, weight resistance exercise for 4 weeks On test day, blood samples were collected at different time intervals: pre-exercise, post-exercise (within 5 min), 3 h post-exercise and 24 h post-exercise of a resistance training session to evaluate the changes in lipid peroxidase and interleukin-8	In the soy group, lipid peroxides levels decreased at 5 min, 3 h and 24 h post-exercise, whereas in the whey group, the decrease was determined at 24 h only. In both groups, a slight increase in interleukin-8 was determined due to exercise-induced muscle stress
*n* = 6 Received sugar-based beverage (30 g of sugar) as a post-training drink every day (Placebo group: *n* = 7 Received a drink similar to soy beverage in both appearance and taste)	4 weeks	Participants performed volleyball training 5 days per week and received supplementation during those days. Pre- and post-intervention changes in the levels of lactate dehydrogenase, creatine kinase, blood urea nitrogen and immunoglobulins	Supplemented group experienced a decrease in creatine kinase as the training proceeded and was significantly lower than that of the other groups after four weeks (*p* < 0.05). No significant differences in blood urea nitrogen were observed

Author,year [reference]	Type of study	Participants	Soy protein-supplemented group
Kritikos et al., 2021 [49]	Randomised double-blind placebo-controlled crossover trial	Male soccer players: average age 21 ± 1.5 years old	*n* = 10 Received 1.5 g/kg/day of soy protein isolate (soy protein: 93 g; carbohydrates: 1 g; fat: 3 g; 379 kcal per 100 g)
Brown et al., 2004 [39]	Randomised double-blind placebo-controlled trial	Experienced weightlifters with at least 1 year or more experience in strength training: age range 19–25 years	*n* = 9 Received micronutrient-fortified soy protein bars (33 g protein/day)
Stroescu et al., 2001 [50]	Randomised placebo-controlled trial	Elite female gymnasts: age average 14.9 ± 1.3 years old	*n* = 7 Received 1 g/kg of Sports Beverage Protein Mix with SUPRO® Isolated Soy Protein

Placebo/comparison group(s)	Study duration	Experimental design	Main outcomes
*n* = 10 Received 1.5 g/kg/day of whey protein isolate (whey protein: 91 g; carbohydrates: 0 g; fat: 1.3 g; 380 kcal per 100 g), (Placebo group: *n* = 10 Received 0.8–1 g/kg/day of isocaloric maltodextrin beverage)	10 days (7 days pre-loading period and 3 days experimental period)	Following a pre-loading that aimed to adjust the total protein intake, two speed-endurance training sessions were performed 1 day apart, over a 3-day experimental period. During each session, muscle damage (delayed-onset of muscle soreness, creatine kinase activity) and redox status (glutathione, total antioxidant capacity, protein carbonyls) were evaluated at baseline (pre-load), following pre-loading (post-load), and during recovery from speed-endurance training Washout period = 1 week	Delayed-onset of muscle soreness, creatine kinase, total antioxidant capacity and protein carbonyls increased, and glutathione decreased equally among trials following speed-endurance training (*p* ≤ 0.05), soy protein supplementation induced a faster recovery of protein carbonyls only at 48 h (*p* ≤ 0.05) compared to whey and placebo groups
*n* = 9 Received micronutrient-fortified whey protein bars (33 g protein/day) (Control group: *n* = 9 Performed training but did not receive any nutritional bars)	9 weeks	Participants were strictly engaged in a strength training program during the study duration. The plasma antioxidant status of participants was measured pre- and post-intervention through analysis of free radical scavenging capacity and myeloperoxidase activity	Comparing the pre- and post-intervention results, the plasma radical scavenging capacities decreases in the whey and control groups, and the values of myeloperoxidase increased in both groups. These values remained unchanged in the soy group
*n* = 7 Received 1 g/kg of placebo (identical in appearance and flavour of test supplement)	4 months	Participants followed a strenuous training programme with daily supplementation. Metabolic parameters were measured: haemoglobin, total proteins, total fats, total cholesterol, free fatty acids, urea, creatinine, glutamic-oxaloacetic transaminase (SGOT), serum glutamic-pyruvic transaminase (SGPT), alkaline phosphatases, calcium, magnesium, immunoglobulins and urine mucoproteins	There was a statistically significant decrease (*p* < 0.01) in alkaline phosphatases and in serum proteins (*p* < 0.05) in the soy group. Changes in haemoglobin and cholesterol were not statistically significant in the supplemented group (*p* > 0.05). Both groups had also an increase in FFAs (*p* < 0.05); the placebo group had higher levels of urine mucoproteins (*p* < 0.05). Both groups found no changes in serum creatinine, calcium and magnesium

*FFAs* free-fatty acids

^*^Indicates the initial number of participants enrolled in the study. The number of participants who completed the study was less

**Table 3:** Summary of randomised controlled trials evaluating the effect of soy-based supplementation on the hormonal response of active individuals and athletes

Author, year [reference]	Type of study	Participants	Soy protein-supplemented group
**Acute supplementation design studies**			
Ghosh et al., 2010 [52]	Randomised double-blind placebo-controlled crossover trial	Male recreational cyclists: age average 21.5 ± 1.1 years	*n* = 8 Received a pre-workout sago-soy protein-based supplement containing 52.5 g of carbohydrates (from sago) and 15 g of proteins (from soy) as a 900 mL beverage

Placebo/comparison group(s)	Study duration	Experimental design	Main outcomes
*n* = 8 Received a pre-workout sago-based supplement containing 60 g of carbohydrates as an 873 mL beverage (Placebo group: *n* = 8 Received a pre-workout placebo drink (873 mL) of no caloric intake)	1 day per study condition	Participants performed a 60 min exercise session on a cycle ergometer at 60% *V*O_2_max followed by a time-to-exhaustion ride at 90% VO_2_max. Insulin levels were measured during and after exercise Washout period = 1 week	Plasma insulin responses in sago-soy and sago supplementations remained at a plateau and increased significantly (*p* < 0.05) after 5 min recovery of exercise

Author, year [reference]	Type of study	Participants	Soy protein-supplemented group
**Chronic supplementation design studies**			
Stroescu et al., 2001 [50]	Randomised placebo-controlled trial	Elite female gymnasts: age average 14.9 ± 1.3 years old	*n* = 7 Received 1 g/kg of Sports Beverage Protein Mix with SUPRO^®^ Isolated Soy Protein
Kraemer et al., 2013 [26]	Randomised double-blind cross-over placebo-controlled trial	Resistant trained males: age average 21.7 ± 2.8 years old	*n* = 10 Received 20 g/day of soy protein isolate as a pre-workout supplement

Placebo/comparison group(s)	Study duration	Experimental design	Main outcomes
*n* = 7 Received 1 g/kg of placebo (identical in appearance and flavour of test supplement)	4 months	Participants followed a strenuous training programme with daily supplementation for the duration of the study. The effect of supplementation on plasma triiodothyronine (T_3_), thyroxine (T_4_), prolactin, estradiol, progesterone, testosterone, and urine 17-ketosteroids-metabolites of androgenic steroids was measured	Soy supplemented group experienced a significant increase in prolactin levels (*p* < 0.01). No major significant differences were observed in other parameters
*n* = 10 Received 20 g/day of whey protein isolate as a pre-workout supplement (Placebo group: *n* = 10 Received 20 g/day of maltodextrin as a pre-workout supplement)	14 days	Participants performed an acute heavy resistance exercise test every day after receiving the supplement for the duration of the study. The effect of supplementation on testosterone, cortisol, estradiol and sex hormone-binding globulin was measured at different time points Washout period = 2 weeks	Soy supplementation slightly, but significantly, reduced testosterone levels after exercise, in comparison with other groups (*p* < 0.05). Whey supplementation resulted in a significant increase in cortisol after exercise in comparison to other groups (*p* < 0.05). Estradiol and sex hormone-binding globulin levels did not differ between the groups or time points

Author, year [reference]	Type of study	Participants	Soy protein-supplemented group
Reidy et al., 2016 [44]	Randomised double-blind placebo-controlled trial	Healthy young recreationally active men: age range 18–30 years	*n* = 23 Received 22 g/day of soy protein-based blend beverage (comprised of 25% protein from soy protein isolate, 25% protein from whey protein isolate, and 50% protein from sodium caseinate)
Berg et al., 2012 [47]	Randomised controlled trial	Clinically healthy sports student: age average 23.6 ± 1.9 years	*n* = 15* Received two 50 g/day servings of soy protein-based supplement (Almased® containing 53.3 g protein, 30.5 g carbohydrates, 2.0 g fat, 354 kcal per 100 g)
Wenxue, 2013 [51]	Randomised placebo-controlled trial	Second grade male volleyball players: average age 20.2 ± 1.9 years	*n* = 6 Received soy protein hydrolysate-based beverage (10 g of soy peptide and 30 g of sugar) as a post-training drink every training day

Placebo/comparison group(s)	Study duration	Experimental design	Main outcomes
*n* = 22 Received 22 g/day of whey protein isolate beverage (Placebo group: *n* = 23 Received22 g/day of isocaloric maltodextrin beverage)	12 weeks	Participants performed 3 days/week of resistance exercise training. They consumed the assigned supplement after exercise (during exercise days) Testosterone level was measured before and after 6 and 12 weeks of resistance exercise training	No significant change in testosterone was observed
*n* = 15* Control group (no supplementation)	6 weeks	Performed moderate endurance training (60 min per day, 5 times a week) at the aerobic threshold. Hormonal parameters were measured before and after the intervention	The supplemented group showed significantly lower differences in insulin in the post-exercise recovery period (*p* < 0.05)
*n* = 6 Received sugar-based beverage (30 g of sugar) as a post-training drink every day (Placebo group: *n* = 7 Received a drink similar to soy beverage in both appearance and taste)	4 weeks	Participants performed volleyball training 5 days per week and received supplementation during those days. Pre- and post-intervention changes in several variables including testosterone levels were measured	The level of serum testosterone in the supplemented group significantly increased compared with that of the control group (*p* < 0.05)

*V*O_2_max, maximum oxygen consumption

*Indicates the initial number of participants enrolled in the study. The number of participants who completed the study was less

**Table 4:** Summary of randomised controlled trials evaluating the effect of soy-based supplementation on the exercise performance of active individuals and athletes

Author, year [reference]	Type of study	Participants	Soy protein-supplemented group
**Acute supplementation design studies**			
Ghosh et al., 2010 [52]	Randomised double-blind placebo-controlled crossover trial	Male recreational cyclists: age average 21.5 ± 1.1 years	*n* = 8 Received a pre-workout sago-soy protein-based supplement containing 52.5 g of carbohydrates (from sago) and 15 g of proteins (from soy) as a 900 mL beverage

Placebo/comparison group(s)	Study duration	Experimental design	Main outcomes
*n* = 8 Received a pre-workout sago-based supplement containing 60 g of carbohydrates as an 873 mL beverage (Placebo group: *n* = 8 Received a pre-workout placebo drink (873 mL) of no caloric intake	1 day per study condition	Participants performed a 60 min exercise session on a cycle ergometer at 60% VO_2_max followed by a time-to-exhaustion ride at 90% VO_2_max. The performance (time-to-exhaustion) of participants was measured as well as their blood urea, ammonia and lactate Washout period = 1 week	Sago-soy supplementation enhanced endurance by 84% (44–140%; *p* < 0.001) and by 37% (15–63%;* p* < 0.05) relative to placebo and sago, respectively. No significant differences in blood urea, ammonia and lactate were observed in the three interventions

Author, year [reference]	Type of study	Participants	Soy protein-supplemented group
**Chronic supplementation design studies**			
Berg et al., 2012 [47]	Randomised controlled trial	Clinically healthy sports student: age average 23.6 ± 1.9 years	*n* = 15* Received two 50 g/day servings of soy protein-based supplement (Almased® containing 53.3 g protein, 30.5 g carbohydrates, 2.0 g fat, 354 kcal per 100 g)
Kritikos et al., 2021 [49]	Randomised double-blind placebo-controlled crossover trial	Male soccer players: average age 21 ± 1.5 years old	*n* = 10 Received 1.5 g/kg/day of soy protein isolate (soy protein: 93 g; carbohydrates: 1 g; fat: 3 g; 379 kcal per 100 g)

Placebo/comparison group(s)	Study duration	Experimental design	Main outcomes
*n* = 15* Control group (no supplementation)	6 weeks	Performed moderate endurance training (60 min per day, 5 times a week) at the aerobic threshold. Physical fitness and exercise-induced stress markers were measured before and after the intervention	Supplementation resulted in a slight increase, yet significant, in running performance and maximum aerobic capacity (2%, *p* = 0.016). The supplemented group also showed significant enhancements in running velocity and decreased lactate values after intervention (− 12%, *p* = 0.003)
*n* = 10 Received 1.5 g/kg/day of whey protein isolate (whey protein: 91 g; carbohydrates: 0 g; fat: 1.3 g; 380 kcal per 100 g), (Placebo group: *n* = 10 Received 0.8–1 g/kg/day of isocaloric maltodextrin beverage)	10 days (7 days pre-loading period and 3 days experimental period)	Following a pre-loading that aimed to adjust the total protein intake, two speed-endurance training sessions were performed 1 day apart, over a 3-day experimental period. During each session, field activity and heart rate were continuously monitored using a global positioning system and heart rate monitors, respectively. Performance (isokinetic strength of knee extensors and flexors, maximal voluntary isometric contraction, speed, repeated sprint ability, countermovement jump), muscle damage (delayed-onset of muscle soreness, creatine kinase activity) and redox status (glutathione, total antioxidant capacity, protein carbonyls) were evaluated at baseline (pre), following pre-loading (post-load), and during recovery from speed-endurance training Washout period = 1 week	Participants experienced a reduction in high-intensity and high-speed running (*p* ≤ 0.05) during speed endurance training in all trials, however, protein supplementations reduced this effect The isokinetic strength, maximal voluntary isometric contraction, 30-m speed, repeated sprint ability and countermovement jump performance of participants were similarly decreased during recovery following speed-endurance training in all trials (*p* ≤ 0.05). 10-m speed was decreased at 24 h only in the placebo trial Delayed-onset of muscle soreness, creatine kinase, total antioxidant capacity and protein carbonyls increased, and glutathione decreased equally in all trials following speed-endurance training (*p* ≤ 0.05), with soy protein supplementation inducing a faster recovery of protein carbonyls only at 48 h (*p* ≤ 0.05) compared to other trials

Author, year [reference]	Type of study	Participants	Soy protein-supplemented group
Laskowski et al., 2003 [53]	Randomised controlled trial	Young judo male athletes: 14–17 years old	*n* = 6 Received 0.5 g/kg/day of soy protein supplement in orange juice
Shenoy et al., 2016 [9]	Randomised double-blind placebo-controlled trial	Trained male boxers (*n* = 20) and road cyclists (*n* = 20): age range 18–22 years old	*n* = 20 Received 25 g × 2 of soy protein supplement (21.1 g of proteins and 21.1 mg of isoflavones genistein per 25 g serving) per day in 300 mL water
Wenxue, 2013 [51]	Randomised placebo-controlled trial	Second grade male volleyball players: average age 20.2 ± 1.9 years	*n* = 6 Received soy protein hydrolysate-based beverage (10 g of soy peptide and 30 g of sugar) as a post-training drink every training day

Placebo/comparison group(s)	Study duration	Experimental design	Main outcomes
*n* = 6 Control group (no supplementation)	4 weeks	Participants performed a Wingate test before and after the intervention to measure the maximal power output and the total work output. The VO_2_max was also measured	Exercise improved the maximum oxygen uptake and Wingate test performance, with a greater effect in the supplemented group. Additional 3 months of training, but without protein supplementation, diminished the effect of the improved VO_2_max
*n* = 20 Received a water-based beverage with artificial sweetener as a placebo	4 weeks	Exercise-induced muscle damage (EIMD) was based on 100 consecutive drop jumps. Pre- and post-intervention highly sensitive C-reactive protein, creatine kinase, and myeloperoxidase were measured. Isometric muscle strength, VO_2_max, heart rate and muscle soreness were also evaluated at baseline (day 1), at 24 h (day 2) and at 48 h (day 3) following EIMD	The efficacy of soy protein supplementation in improving the recovery process, with respect to inflammatory and muscle damage markers, was depicted by a greater decrease in the mean values of boxers only. Soy protein supplementation was effective in decreasing muscle soreness and enhancing recovery after 48 h compared with placebo
*n* = 6 Received sugar-based beverage (30 g of sugar) as a post-training drink every day (Placebo group: *n* = 7 Received a drink similar to soy beverage in both appearance and taste)	4 weeks	Participants performed volleyball training 5 days per week and received supplementation during those days. Pre- and post-intervention changes in body compositions, RPE grade and biochemical indices were measured	Peptide supplementation significantly increased the mass and the lean body mass of the peptide group at the end of the intervention (*p* < 0.05); however, the rating of perceived exertion grade of the peptide group decreased significantly (*p* < 0.01) as well as creatine kinase (*p* < 0.05). No significant changes were observed in lactate dehydrogenase levels

*VO*_*2*_*max* maximum oxygen consumption, *RPE* rated perceived exertion

*Indicates the initial number of participants enrolled in the study. The number of participants who completed the study was less

### Risk of Bias Assessment

Two reviewers (D.S. and R.Z.) independently assessed the risk of bias within the included studies using the Cochrane’s revised risk of bias tool, RoB2 [32]. A third reviewer (A.AR.) arbitrated when needed. The following biases were considered: randomisation process, period and carryover effects (only for crossover studies), deviations from interventions, missing outcome data, outcome measurement and selection of the results. Each domain was judged singularly as low, with some concerns or high risk following the rules of RoB2 decision trees. The study was classified as low risk if a low risk of bias for all domains was demonstrated, and a high risk of bias if it was demonstrated a high risk in at least one domain. The ‘some concerns’ overall judgment followed the same rules of the high risk, respectively [32]. Bias results were printed by using the web app *robvis* [33].

## Results

### Study Selection

**Fig. 1:**
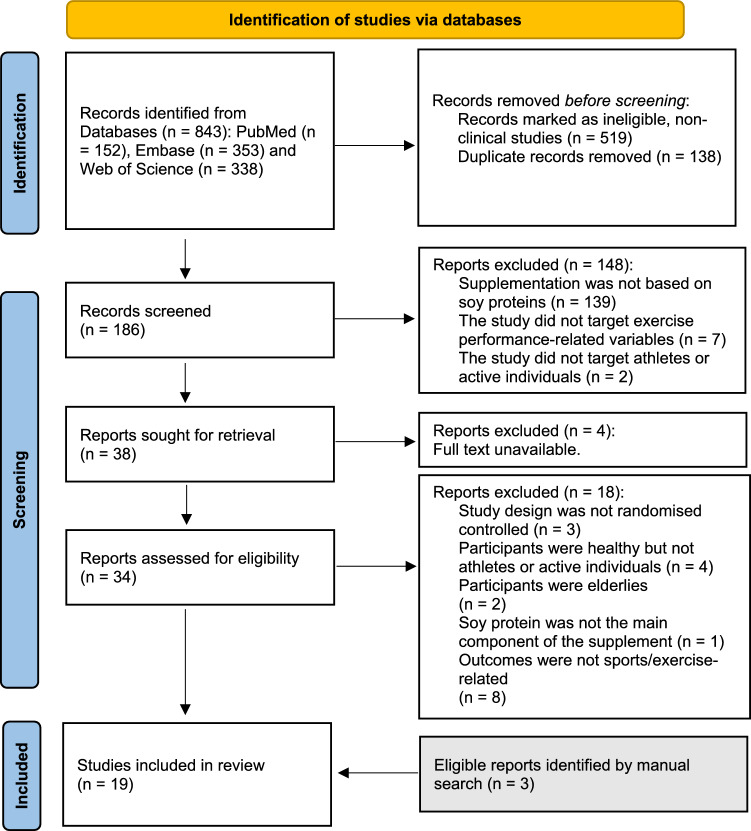
Preferred Reporting Items for Systematic Reviews and Meta-Analyses (PRISMA) flow diagram

The review identified 840 records by searching the three databases. Clinical-based research articles were identified (*n* = 186). The clinical-based studies were screened by title, abstract and keywords by two of the reviewers (R.Z. and A.AR.) independently. A third reviewer (S.G.) arbitrated when needed. A total of 148 articles were excluded. A total of 38 articles were sought for retrieval, of which *n* = 4 were excluded since the full text was not available. The remaining 34 articles were then assessed for eligibility, of which 18 were excluded. A total of three eligible articles were identified through a manual search. Finally, a total of 19 articles were determined as eligible articles and were included in the qualitative synthesis of the current systematic review. The details of the study selection process are shown in Fig. 1. Some studies were excluded because they did not follow randomised controlled trial design, although they investigated relevant outcomes such as the effect of SP on skeletal muscle volume and strength [34], muscle protein synthesis [35] and muscle mass and strength [36]. Some studies did not specify the physical activity status of the participants or were not physically active, yet were relevant and investigated the effect of SP on lean body mass [37], lean tissue mass, muscle strength [25, 27] and sex hormones [38].

### Characteristics of the Included Studies

The studies were classified on the basis of the topic of the study and relevant outcomes. The four areas considered were muscle adaptations, metabolic/blood circulating biomarkers and antioxidant status, hormonal response, and exercise performance. Some studies investigated more than one area.

A total of nine studies were categorised under muscle adaptations [17, 39–46]. A total of 314 individuals were the participants in these studies, from which 8 participants were engaged in resistance training and 27 participants were experienced weightlifters. The remaining individuals were recreationally active.

Six studies were categorised under metabolic/blood circulating biomarkers and antioxidant status [39, 47–51]. A total of 118 individuals participated in these studies, which included weightlifters (*n* = 27), volleyball players (*n* = 19), resistant-trained individuals (*n* = 18), elite gymnasts (*n* = 14), soccer players (*n* = 10), and sports students (*n* = 30).

A total of six studies were categorised under hormonal response [26, 44, 47, 50, 52]. A total of 149 individuals was the sum of participants in these studies, from which 14 were elite female gymnasts, 10 were resistant trained males, 8 were cyclists, 19 were volleyball players, 68 were recreationally active men and 30 were sports students.

A total of six studies explored exercise performance-related outcomes [9, 47, 49, 51–53]. The sum of the participants in these studies was *n* = 119, which included male cyclists (*n* = 28), boxers (*n* = 20), volleyball players (*n* = 19), judo athletes (*n* = 12) and soccer players (*n* = 10). All the athletes involved in the exercise performance-related studies were male. Only one study that targeted sports students involved both genders (*n* = 30; 20 males and 10 females).

### General Findings

#### Muscle Adaptations

In this systematic review, five studies explored the impact of SP versus whey protein pertinent to lean mass or muscle growth and muscle strength. In one study, consumption of 19 g of whey protein isolate or 26 g of SP isolate in 48 young untrained individuals who underwent supervised weight training 3 × per week for 12 weeks did not elicit any significant differences between groups [41]. Lean body mass and vastus lateralis thickness changes did not differ between interventions, although a mean small but insignificant reduction in vastus intermedius thickness was observed in the soy (− 0.10 ± 0.98 cm) versus the whey protein group (0.01 ± 0.12 cm), which may not be clinically meaningful particularly after considering the high standard deviation values presented. Likewise, no significant changes were observed for peak torque flexion and extension between interventions, although whey exhibited more positive outcomes (extension: 30.5 ± 15.6 Nm versus 19.7 ± 15.4 Nm; flexion: 14.2 ± 8.7 Nm versus 11.4 ± 12.9 Nm). It is worth mentioning that both groups consumed a nutrient-matched diet (containing a dietary protein content of 1.3–1.4 g/kg body weight (BW)/day (grams per kilogram of body weight per day). Similarly, daily consumption of micronutrient-fortified SP or whey protein bars containing 33 g protein did not elicit significant differences in lean body mass between groups, following a 9-week weight training protocol in young male weightlifters [39]. However, data on the nutrient intake to account for the impact of diet in this cohort was not presented.

Furthermore, in young, recreationally active men that were given the option of drinking macronutrient-matched fat-free milk or fat-free SP and trained 5 × per week for 12 weeks on a resistance exercise program, notable changes in body composition were observed [40]. In particular, although increases in muscle fibre type II area, as well as fat- and bone-free mass, were displayed in all groups, participants consuming milk displayed significantly greater benefits compared with soy and control groups. In this study, the milk group ingested a slightly greater, but insignificant, protein intake at week 6 (1.8 g/kg BW/day versus 1.7 g/kg BW/day) and at the end of the intervention (1.8 g/kg BW/day versus 1.6 g/kg BW/day).

Lastly, when SP (25 g/day) was combined with dairy during a 3 × per week 12-week resistance training and macronutrient-matched protocol in young men, no significant changes in knee extensor muscle thickness, whole-body lean mass, squat 1-RM, knee extension strength, and chest press were observed versus whey, while consuming identical dietary protein intake at week 6 (whey protein group: 1.54 ± 0.11 g/day; soy-dairy blend: 1.68 ± 0.10 g/day) and at week 12 (whey protein group: 1.64 ± 0.11 g/day; soy-dairy blend group: 1.54 ± 0.10 g/day) [44]. In a subsequent study including, in part, the same participants, the authors also showed that all groups demonstrated identical leg muscle hypertrophy and vastus lateralis myofibre type-specific cross-sectional area [46].

Four studies considered investigating acute supplementation of SP on muscle adaptations [17, 42, 43, 45]. In young healthy individuals engaging with resistance exercise, an 18 g of SP beverage resulted in a lower net muscle protein balance versus milk protein consumption, as seen via fractional synthetic rates (milk protein: 0.10 ± 0.01%/h; SP: 0.07 ± 0.01%/h; *p* = 0.05) [43]. In addition, young recreationally active men who consumed whey protein versus SP (20 g) with free leucine had significantly higher post-prandial peak plasma leucine concentrations compared to SP (*p* < 0.05), although statistically, myofibrillar and mitochondrial synthetic rates over 360 min post-concurrent exercise did not differ between treatments (*p* > 0.05). Protein signalling through mTORC1^Ser2448^, p70S6k^Thr389^, 4E-BP1^Thr37/46^, and rpS6^Ser235/236^ did not differ between groups [17]. In a similar cohort, the amount of phenylalanine transported into muscle and the mRNA expression of specific amino acid transporters, namely system L amino acid transporter 1, solute-linked carrier (SLC) 7A5, CD98/SLC3A2, system A amino acid transporter 2, system A amino acid transporter 2, proton-assisted amino acid transporter 1, and cationic amino acid transporter 1, were found to be significantly higher (*p* < 0.05) following a soy–dairy protein blend (20 g) versus whey isolate. However, compared with those who were supplemented with whey protein, the soy–dairy protein blend led to a considerably longer and more favourable net phenylalanine balance (*p* < 0.05) during post-exercise recovery [42]. An identical cohort and treatment by Reidy et al*.* (2013) showed that in comparison with whey protein supplementation, SP-based blend led to a slower initial increase of plasma branched-chain amino acids and maintained higher levels during post-exercise recovery (*p* < 0.05). After exercise, the fractional synthesis rate of mixed-muscle protein was increased in both groups, although it was still considerably higher in the soy blend group (soy–dairy blend group: 0.087 ± 0.003%/h; whey protein group: 0.074 ± 0.010%/h) (*p* < 0.05). Finally, both groups led to significant increases in mTORC1 signalling, but whey protein did not enhance S6K1 phosphorylation at 300 min post-exercise (*p* > 0.05) [45].

#### Metabolic and Antioxidant Status

In our review, six articles were selected for their metabolic/blood circulating biomarkers and antioxidant insights [39, 47–51]. Three of them were specifically mentioned to be double-blind studies, and only one was a crossover trial. Apart from one research study that lasted 4 months [50], most of the studies were conducted for 4–9 weeks, and one study lasted for 10 days (7-day pre-loading period and 3-day experimental period) [49].

Generally, participants were young adults between the ages of 20 and 24. The number of subjects varied from 6 to 15, with neither sex always being represented. All of the participants had at least 6 months of sports exercise experience and their physical activities ranged from endurance training to gymnastic practises to soccer and volleyball. In most studies, beverage supplements were cited as a prevalent source. Indeed, only one trial used protein bars. Several athletes took protein supplements before or after training, and their daily SP intake ranged from 10 g [51] to 90 g [49]. It is important to mention that the study by Wenxue, 2013 [51], used soy peptides rather than soy proteins. The soy peptides mainly comprised a pentapeptide (Leu-Ala-Pro-Glu-Glu), hexapeptide (Met-Ser-Leu-Pro-Thr-Asn) and octapeptide (Arg-Leu-Met-Leu-His-Leu-Ala-Pro).

Different measures were used to evaluate the metabolic pathways with distinct results. In a study, moderate resistance training with an SP supplementation resulted in a reduction of exercise-induced elevations in blood circulating biomarkers (e.g. triglycerides and uric acid) and a decrease in lactate levels (− 12%, *p* = 0.003) [47]. Further, soy peptides were also investigated to reduce creatine kinase serum levels after a month [51] and alkaline phosphatases after 4 months of treatment [50] compared with placebo.

When Kritikos et al*.* (2021) looked at antioxidant capacity, they found that protein carbonyls, as potential indicators of oxidative stress, tended to be recovered faster with SP supplementation rather than with whey protein or isoenergetic placebo only 48 h after the initial speed-endurance exercise; on the contrary, glutathione decreased equally among trials [49]. It is important to highlight again that the supplementation period in this study (10 days [7-day pre-loading period and 3-day experimental period)] was relatively shorter than all the other studies, thus this needs to be taken into consideration. In another study, the soy group that was given a supplement of 40 g/day before a moderate weight resistance workout, had lower values for serum lipid peroxides from 5 min until 24 h after the workout compared with the whey protein group (40 g/day) [48]. Also, after 9 weeks of strength training, plasma radical scavenging capacities fell in both whey and control male weightlifter groups; however, the consumption of 33 g of SP each day avoided a decline in antioxidant capabilities [39].

While contemplating inflammation markers, in both the soy and whey groups, a small rise was seen for interleukin-8, a chemokine that induces angiogenesis in working muscles during exercise, which was consistent with the idea that the exercise session induced moderate muscle stress [48]. In another clinical trial, muscle and systemic stress indicators were elevated; especially for high-sensitivity C-reactive protein (hs-CRP) that was significantly elevated in both soy-supplemented and untreated groups, the day after the field test (*p* = 0.013) [47]. Finally, myeloperoxidase levels, an enzyme linked to inflammation and oxidative stress, were found to increase in both the whey and training-alone groups, but there were no significant differences with the SP group [39].

#### Hormonal Response

Five relevant investigations on athletes were found, regarding the hormonal assessments following SP supplementation [26, 44, 47, 50, 52]. Only one acute study (1 day per condition) was identified [52], while four studies performed a chronic supplementation period ranging from 14 days to 4 months. The timing of supplement intake differed between studies, at baseline and during steady-state exercise [52], before exercise [26] or after exercise [44, 50]. One study did not mention the exact timing of supplement intake [47]. In addition, a sixth study [51], which focussed on metabolism and exercise performance, considered measuring testosterone levels as a secondary variable. This study lasted for 4 weeks (chronic supplementation) and supplement intake was assigned to be after training. The supplement was based on soy protein peptides as described in the previous section.

In 2001, Stroescu et al*.* (2001) conducted a randomised double-blind placebo-controlled trial, in which for 4 months 14 Olympic female gymnasts were supplemented with 1 g/kg BW/day SP or with placebo composed of 10 g of sugars and 3 g of cocoa [50]. After the intervention period, the supplemented group experienced an increase in lean body mass, and serum levels of prolactin and T_4_, but a decrease in serum alkaline phosphatases. On the contrary, the non-supplemented group had a decreased level of serum T_4_ and an increased level of urinary mucoproteins.

In a 6-week randomized crossover trial, a pool of ten resistance-trained men were supplemented with 20 g of whey protein isolate, SP isolate or an isoenergetic maltodextrin-based placebo [26]. It was reported that 2 weeks of SP supplementation lowered serum testosterone, while whey protein altered the response of cortisol by reducing its increase during recovery. In contrast, another study observed that a 12-week resistance exercise training did not alter testosterone levels in active men after supplementation with 22 g/day of SP-based blend beverage, whey protein, or a placebo [44]. Nevertheless, the supplementation of 10 g/day of soy peptides for 4 weeks resulted in a significant increase in testosterone levels in volleyball players [51].

In relation to insulin, the data were consistent. The plasma insulin response was raised compared with placebo following acute SP supplementation and soy with carbohydrates after 5 min of recovery from an endurance cycling exercise [52], as well as after moderate endurance training with more evidence in the placebo group [47].

#### Exercise Performance

Six studies met the inclusion criteria of this systematic review [39, 47–51], from which two studies were planned as a crossover design [49, 52]. Only one study compared SP supplementation with whey protein isolate [49]. This study set the protein intake to 1.5 g/kg BW/day for all subjects consuming either whey or soy protein.

Supplementation period differed from 1 day [52] to 6 weeks [47]. Supplementation protocols applied either a single bolus dose [49, 51, 53] or at three points during the steady-state exercise [52]. The supplement dosage ranged from 10 [51] to 53.3 g/day [47]. It is important to mention that the study by Wenxue, 2013 [51], used soy peptides rather than soy proteins.

Four study protocols included chronic exercise programme lasting between 4 and 6 weeks [9, 47, 51, 53]. Two studies applied acute muscle-damaging training including a 60-min steady-state exercise session at 60% of VO_2_max, followed by a time-to-exhaustion ride at 90% of VO_2_max in recreational cyclists [52] and two speed endurance training (~ 60 min including one set of eight (30-s each) maximum-intensity repetitions with a passive recovery of 2.5 min) performed one day apart in professional soccer players [49]. Three studies determined the effect of SP supplementation compared with placebo [9, 53] or a control group that did not consume any supplement [47]. All of the three studies included long-term training along with the SP supplementation. A study on highly-trained boxers and road cyclists indicated that 4 weeks of soy isolate supplementation resulted in a decline in the reduction of isometric muscle strength and muscle force following exercise-induced muscle damage, and a quicker recovery (assessed by examining mean isometric peak torque, and blood hs-cRP, and creatine kinase levels at baseline, at 24 h and at 48 h after exercise) of flexors and extensors of both limbs in the boxer group only, not in the cyclists [9]. A study on professional junior judoists also found positive results, namely higher aerobic power (assessed via VO_2_max) and anaerobic capacity (determined via Wingate test) after 6 weeks of SP supplementation compared with the placebo group [53]. Another study also indicated a slight improvement in running performance (2%, *p* = 0.016), running velocity at both 2 mmol/L and 4 mmol/L thresholds (+ 15%, *p* = 0.011), and lower lactate concentrations (− 12%, *p* = 0.003) after 6 weeks of SP supplementation along with moderate endurance training (60 min/day for 5 days per week) [47].

Two studies investigated the effect of acute [52] and chronic [51] SP co-administered with carbohydrates. One of the studies on professional male volleyball players compared soy peptide (10 g) + sugar (30 g), sugar (30 g) or placebo for 4 weeks along with training at heavy loads. The findings showed that perceived exertion significantly decreased in the soy group compared with placebo; however, results did not differ compared to the sugar group [51]. The other study on male recreational cyclists compared sago starch (60 g), sago + soy (52.5 g CHO + 15 g SP), and a placebo drink (acute supplementation). The sago + soy group showed higher endurance performance compared with the sago-only and placebo group (by 37% and 84%, respectively) [52]. Only one study compared the effect of SP on exercise performance with whey protein [49]. It showed that both whey protein and SP supplementation following speed-endurance training in highly trained soccer players caused an increase in high-intensity running and high-speed running compared with the placebo group. Neither whey protein nor SP supplementation affected the average fatigue index (%), blood lactate concentrations, maximal voluntarily isometric contraction, countermovement jump height or repeated sprint ability [49]. It is important to highlight again that the supplementation period in this study [10 days (7 days pre-loading period and 3 days experimental period)] was relatively shorter than all the other studies, thus this needs to be taken into consideration.

### Sub-Group Analysis

Considering the variability of participants engaging with exercise (i.e. recreationally active, professional athletes, resistance trained or untrained individuals), we could not extrapolate definitive conclusions related to each group in regard to muscle adaptations, metabolic and antioxidant status, hormonal response and exercise performance, highlighting that our findings may be pertinent to generally active individuals only. In addition, with respect to sex differences, a previous meta-analysis has reported no effect of sex on response to protein supplementation [54].

### Risk of Bias in Studies

**Fig. 2:**
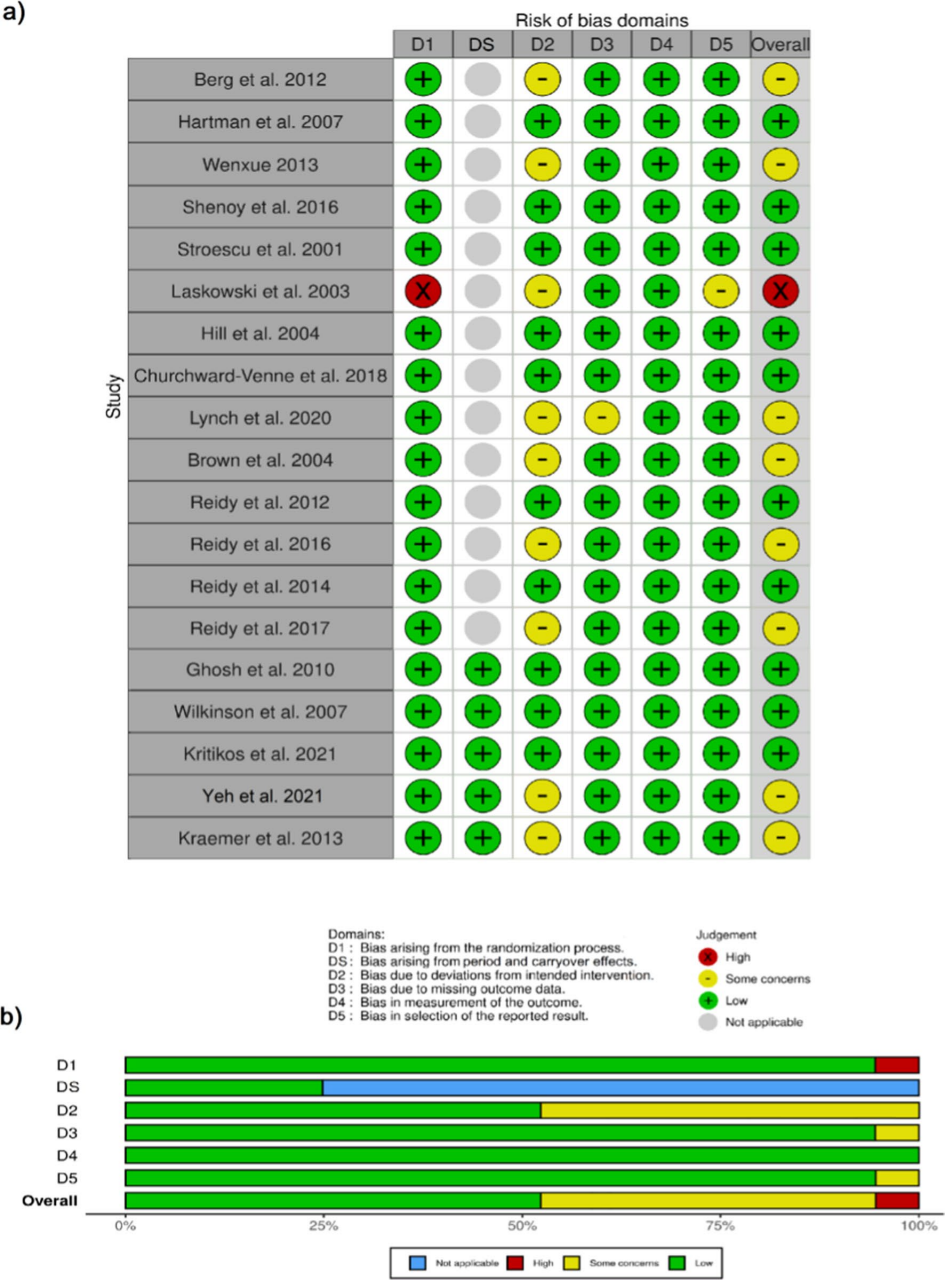
Assessment of bias of the randomised studies. **a** Traffic light plot and **b** summary plot

The analysis of bias concluded that half of the studies had a low risk overall (Fig. 2). The main concerns were raised for the risk of deviation from the intended intervention, which was judged to have a medium risk (some concerns) in nine studies [26, 39, 41, 44, 46, 47, 51, 53, 55]. This was mainly due to the lack of supervision on physical activity and dietetic protocol compliance in long-term studies, while shorter studies had fewer concerns. Missing data was also a concern in one study [41] since a significant dropout percentage was reported and no evaluation of attrition bias not intention-to-treat analysis was reported. The risk of bias for the measurement outcomes was generally low. Low bias was associated with reporting results in all studies with an exception of one study [53] due to additional analyses not declared in the methods section. A high risk of bias was detected in the randomisation process of the same randomised controlled trial study, because it did not blind the intervention between groups. All crossover studies [26, 43, 49, 52, 55] included a reasonable wash-out period between the interventions; thus, the carryover risk was set as low.

## Discussion

### Muscle Adaptations

Based on our findings, whey displayed slightly favourable outcomes pertinent to lean body mass and peak torque compared to SP during suboptimal amounts of protein (1.3–1.4 g/kg BW/day) following resistance exercise in young adults [41]. Utilizing higher dietary protein regimes (~ 1.6 to 1.8 g/day), Hartman et al*.* (2007) showed that milk consumption led to increased levels of muscle fibre type II [40]; however, no changes in knee extensor muscle thickness, whole-body lean mass, squat 1-RM, knee extension strength, chest press, and vastus lateralis myofibre type-specific cross-sectional area between soy and whey protein arms were observed [44, 46].

Research has suggested a 1.6 g/kg BW/day protein intake as the optimal dose for muscle building in healthy individuals [54]. The aforementioned findings suggest no consistent differences in body composition and strength parameters between soy and whey protein supplementation with increased total dietary intakes (≥ 1.6 g/kg BW/day); however, during suboptimal intake (e.g., 1.3–1.4 g/kg BW/day), a slightly greater effect in favour of whey was observed. These findings correspond with a non-randomized trial in physically active young individuals consuming 1.6 g/kg BW/day protein, in which both whey and SP supplemented arms of omnivorous and vegan groups, respectively, demonstrated identical improvements in leg lean mass, rectus femoris and vastus lateralis cross-sectional area, and leg press 1RM, after a 12-week resistance exercise protocol [36]. Considering that whey protein exhibits a more favourable amino acid profile compared with soy protein, their slight differences are compensated at slightly higher intakes.

Several studies have taken an in-depth look that may describe the benefits pertinent to both types of protein ingested and their modest differences in muscle-related outcomes. Mechanistically, in a double-blind manner, Reidy et al*.* (2014) looked at recreationally active young individuals at rest, following acute resistance exercise, and after ingesting either a (soy–dairy) protein blend or whey protein (acute supplementation) for one hour after exercise, attempting to measure amino acid transport and transporter expression in their skeletal muscle [42]. Both groups experienced an increase in phenylalanine transport into muscle and mRNA expression of a subset of amino acid transporters. In contrast to whey protein, the protein blend caused a prolonged and favourable net phenylalanine balance during post-exercise recovery, whereas both groups experienced similar increases in myofibrillar protein synthesis following exercise (at 0–4 h). Therefore, while the effects of post-exercise protein intake on AAT expression, transport into muscle, and myofibrillar protein synthesis were improved by both protein sources, the effects of the (soy–dairy) protein blend on net amino acid balance across the leg were marginally longer than those of whey protein; at 5 h, the SP blend led to a more sustained rise in myofibrillar protein synthesis and S6K1 signalling [45]. Nevertheless, dairy is comprised in part of whey, hence, accurate conclusions on the exact impact of soy should not be extrapolated. The same authors also found similar effects on myonuclei content in vastus lateralis [46] and measures of lean mass and muscle strength between supplements, as previously mentioned [44]. Conversely, examination of a nutrient-matched beverage of soy or milk (18 g protein; acute supplementation) after resistance exercise in healthy resistance-trained young men demonstrated a higher muscle fractional synthesis rate in muscle following milk consumption (0.10 ± 0.01%/h) compared with soy (0.07 ± 0.01%/h; *p* = 0.05) [43], which may be ascribed to the more competent properties of milk’s amino acid profile during suboptimal intakes. Added to this, in a non-randomised controlled trial, resistance trained men consuming 10 g essential amino acids in the form of whey hydrolysate or SP isolate after unilateral leg resistance exercise, elicited differing results pertaining to mixed muscle protein synthesis [35]. Specifically, whey consumption led to a 31% greater mixed MPS rate compared with SP post-exercise, although similar values were depicted at baseline (*p* = 0.069). These findings, however, were based on a period of 180 min following exercise. Nevertheless, another double-blind randomised controlled trial comprised of recreationally active young men ingesting 45 g of carbohydrates and 20 g of whey or soy (acute supplementation) followed by concurrent exercise found that the whey group had substantially greater postprandial peak plasma leucine concentrations than the SP group, although no differences were observed in myofibrillar and mitochondrial protein synthesis within 360 min post-recovery [17]. In addition, mTORC1^Ser2448^, p70S6k^Thr389^, 4E-BP1^Thr37/46^, and rpS6^Ser235/236^ signalling was similar between groups.

Taken together, SP supplementation may be an effective alternative to whey in promoting optimal muscle mass and strength gains, at least in young athletic populations, utilizing a protein intake of ≥ 1.6 g/kg BW/day. In our opinion, this is most likely because at such a high dose, the amino acid availability is being compensated in comparison to whey protein. However, this observation was based on a limited number of trials (*n* = 2), from which the one study that showed identical results used soy–dairy blend supplement [44].

Overall, the possible mechanisms of action of SP supplementation in improving muscle adaptations could be linked to different processes. For instance, SP isolate is a rich source of leucine with a content of 8.0 g/100 g of protein [56], which can effectively activate the mTOR signalling pathway and muscle protein synthesis [57]; this effect becomes stronger when leucine-rich protein products are consumed after resistance exercise [57]. Lastly, it has been reported that SP and its peptides can enhance the sensitivity of pancreatic β cells, promoting insulin secretion [58] that can increase the cellular uptake of amino acids and stimulate muscle protein synthesis [59, 60].

### Metabolic and Antioxidant Status

SP supplementation showed promising results in terms of downregulating oxidative stress parameters in plasma in comparison with whey protein and placebo. Two long-term studies (4 and 9 weeks) comparing SP versus whey protein on metabolic/blood circulating biomarkers and antioxidant status revealed that consuming whey or SP matched for nitrogen content (33–40 g/day) decreased serum lipid peroxides after moderate weight exercise training in recreationally trained young men compared with the whey group [48] and improved plasma radical scavenging capacities and downregulated myeloperoxidase concentrations in trained weightlifters compared with the whey and placebo groups [39]. In addition, one acute study determining the efficacy of whey versus SP (1.5 g/kg BW/day) on blood circulating biomarkers and antioxidant status showed that although total antioxidant capacity and creatine kinase levels stayed elevated after speed endurance training regardless of the supplementation group, plasma protein carbonyls were lower in the soy group (*p* < 0.004) and tended to be lower in the whey protein group (*p* < 0.061) compared with the placebo group [49]. The potential mechanism behind these results can be associated to a longer intervention time required to recognise the potential efficacy of SP on antioxidant capacity against the exercise-induced oxidative stress. It is stated that isoflavones and saponins can be effective bioactive molecules with their conservative roles on DNA oxidative damage and their effects on increasing plasma antioxidant capacity [61]. Given these findings, long-term SP supplementation may create meaningful effects on antioxidant status by down-regulating plasma oxidative stress parameters with its antioxidant content including isoflavones and saponins [62], consistent with previous research [63]. However, as there is only one acute study of soy protein supplementation and antioxidant status, more studies are needed to elucidate the potential impact of acute studies.

It is well known that exercise intensity and duration are potent determinants of exercise-induced oxidative stress. One study found no meaningful differences between a SP supplementation (53.3 g protein/day) and an exercise only group in terms of muscular stress indicators (lactate dehydrogenase, creatine kinase, myoglobin levels) and systemic and immunologic stress indicators (serum hs-CRP, interleukine-6 and interleukine-10) [47]. They suggested that these results could be attributed to the duration of stress test (60 min), which might be not enough to induce oxidative stress. Only two long-term studies involved high-intensity training which is known to further trigger exercise-induced oxidative stress [64]. One study on elite female gymnasts showed that 4 months of SP supplementation (1 g/kg BW/day) along with strenuous training caused a meaningful increase in serum IgA (a major component of the humoral immune system), and lower urine mucoprotein levels (a biomarker of metabolic stress), compared with the control group through strenuous training alone. Another study on male trained volleyball players revealed that lower creatine kinase levels were observed in the SP group (10 g SP + 30 g sugar) after 4 weeks of strenuous training compared with the sugar (30 g sugar only) and the placebo group; however, another acute study involving strenuous training resulted in similar creatine kinase concentrations following speed endurance training in the whey and soy protein groups (1.5 g/kg BW/day) [49]. Thus, soy peptides can protect cell membrane and muscle tissue damage during strenuous exercise by reducing creatine kinase leakage from muscle cells [65]. However, it may require a long exercise period to show this potential effect. Further research is needed to clarify these results.

Another point to consider is that soy-derived bioactive peptides are also suggested for their immunomodulatory roles by its immunostimulant Q (Gln)-abundant peptides [66]. In addition, research addressed that soy peptides might strengthen immune system during negative nitrogen balance [67]. Since negative nitrogen balance is a well-known condition during exercise, soy supplementation might provide several beneficial effects on immunoregulation [68]. Moreover, some soy peptides generated upon digestion, such as Arg-Gln-Arg-Lys and Val-Ile-Lys, have been reported for anti-inflammatory activity [69]. It is well known that inflammation can impair muscle protein synthesis [70]; soy peptides with anti-inflammatory activity may indirectly support muscle protein synthesis by reducing inflammation. Further studies are needed to clarify the presence of these interaction in athletes/active individuals.

In general, long-term SP supplementation with exercise training may provide benefits on exercise-induced oxidative stress by preventing excessive accumulation of oxidative parameters such as lipid peroxides and myeloperoxidases, which can further increase exercise-induced oxidative stress compared with the whey protein as matched for the nitrogen content [39, 48, 50, 51]. Although these benefits are often attributed to its antioxidant properties, further studies involving next-generation sequencing techniques such as omics could greatly contribute to our understanding of exactly how and which subtype of SP acts as an antioxidant against exercise-induced oxidative stress and muscle damage.

### Hormonal Response

Studies mainly focused on insulin response after exercise and sex hormone alteration after soy supplementation. The studies evaluating the efficacy of soy protein on hormonal response declared conflicting results. While two studies showed an increase in insulin concentration after exercise in the soy group compared with the CHO only and the placebo group [52] and the control group (no supplementation) [47], and one study indicated no difference between soy and whey protein groups [44]. Insulin generally increased during recovery when SP or SP co-administered with CHO were given in pre-/during exercise compared with placebo or control group with no supplementation [47, 52]. It has been reported that SP is capable of improving the sensitivity of pancreatic β cells and stimulating insulin secretion due to the structural pattern of SP and the presence of key amino acids (e.g., glycine and arginine) in its composition [58].

Female athletes supplemented with SP experienced an increase in prolactin and T_4_ serum levels [50]. T_4_ hormone regulates how the body uses energy (i.e. storage and expenditure) which may influence physical activity [71]. As a thyroid hormone, it may also influence skeletal muscle development and muscle regeneration [72].

Regarding sexual hormones, it is still rather unclear whether they are significantly affected by soy versus whey protein consumption, even though estrogenic signalling may be potentiated via SP supplementation. Indeed, among soy constituents, isoflavones are the phytoestrogens that may inhibit aromatase enzymes, thereby increasing estradiol production and exerting estrogenic effects in humans [73, 74]. However, the exact amount and time frame necessary for SP to achieve these potential effects have not been definitively investigated. Also, in non-athletic healthy men supplemented with different combinations of SPs and whey in conjunction with a resistance-training program, the testosterone/estradiol ratio increased, and estradiol decreased across all groups. Lending support to previous claims, within-group analysis revealed that soy supplementation resulted in significant increases in the testosterone/estradiol ratio [38]. In an analogous excluded study conducted on college-aged men following 12 weeks of full-body resistance exercise training, serum estradiol concentrations were not significantly altered by SP supplementation; however, serum total testosterone concentrations increased only in the whey protein group [75]. A study found that supplementation with SP combined with resistance exercise for 12 weeks had no effect on testosterone levels in physically active young men [44], while soy peptides significantly increased testosterone levels in volleyball players after 4 months of supplementation [51]. More studies on athletes are required to substantiate these data and explore soy peptides.

### Exercise Performance

All studies suggested beneficial effects of SP supplementation on exercise performance. Studies with acute soy supplementation suggested that SP supplementation along with CHO (15 g SP, 52.5 g CHO) during steady state exercise may provide benefits by improving endurance in recreational cyclists compared with both the CHO-only and placebo groups [52]. Both whey and soy protein supplementation improved high-intensity and high-speed running performance in well-trained soccer players compared with the placebo group when daily protein intake set as 1.5 g/kg BW/day [49]. Studies with SP supplementation for 4–6 weeks indicated that 4 weeks of SP supplementation may be beneficial for exercise performance by decreasing perceived exertion after training at heavy load in trained volleyball players (10 g soy peptide + 30 g CHO/day) [51], by enhancing aerobic power and anaerobic capacity in professional junior judoists (0.5 g/kg BW/day of SP) (Laskowski & Antosiewicz, 2003), and by improving isometric muscle strength and muscle recovery after EIMD exercise in trained boxers (42.2 g SP/day) [9, 52, 53]. In addition, the only study on non-athletes also showed that a 6-week SP supplementation combined with moderate endurance training increased running velocity and decreased lactate accumulation in healthy sports students compared with the control group with no supplementation [47].

These favourable responses after SP supplementation in young athletes and non-athletes are consistent with literature determined the effects of SP on young adults [17, 25]. An excluded study by Candow et al. (2006) [25] applied either whey or SP (1.2 g/kg BW/day) for 6 weeks combined with resistance training in young untrained adults. The findings showed that both protein supplementation provided minimal positive effects on muscle mass and strength compared with the placebo group. In addition, another randomised controlled trial conducted by Churchward-Venne et al. (2019) [17] showed that whey, soy or leucine-enriched SP, along with carbohydrate intake, are similar in muscle protein synthesis rates after resistance and endurance exercise, indicating co-ingestion of CHO with 20 g SP provides similar benefits in myofibrillar and mitochondrial protein synthesis rates in young healthy non-athlete recreationally active men compared with whey and leucine-enriched SP. These results indicated that 4 to 6 weeks of SP supplementation may provide benefits in improving exercise performance by improving maximal cardiac output, decreasing perceived exertion, enhancing isometric muscle strength and improving running velocity at 2 and 4 mmol lactate thresholds in young adults. However, studies included this section administered a wide range of SP doses to the participants during the study period, and only one study set daily protein intake as 1.5 g/kg BW/day, as recommended for athletic population [49]. As total daily protein dietary intake may affect the indicators of exercise performance [76], these findings need to be interpreted with caution.

Two crossover studies investigated the acute impact of SP on exercise performance compared with the placebo group [49, 52]. One study on male recreational cyclists applied either sago starch, sago + SP or placebo during 60 min of steady-state cycling, followed by a time to exhaustion ride at 90% *V*O_2_max. Findings indicated that sago + SP supplementation can delay fatigue, thus improve endurance performance compared with both sago starch only and the placebo groups [52]. Another study comparing the efficacy of SP with whey protein and placebo on recovery following speed endurance training in competitive male soccer players showed that both whey and SP supplementation groups showed better high-intensity and high-speed running. The findings showed that SP can be as effective as whey protein on recovery kinetics if the daily protein intake is adjusted to 1.5 g/kg BW/day [49]. Both whey and soy protein are classified as high-quality proteins, based on the Digestible Indispensable Amino Score (DIAAS), with an average DIAAS score of ≥ 75 [77]. Studies on this protein mostly adjusted protein intake as either a total supplementation dose applied (e.g., 1.2 g/kg whey or 1.2 g/kg SP) [25], or leucine-matched protein supplementation (e.g., 19 g whey protein isolate versus 26 g SP isolate) [41], generally focussed on their efficacy in muscle protein synthesis and yielded controversial results. These controversies may be due to the study protocol applied including exercise type, duration, supplementation dose/time/duration and study population [78–80]. In addition, a longer training intervention lasting ≥ 12 weeks has been suggested to observe the exact impact of protein type on muscle protein synthesis and strength [25]. Although muscle protein synthesis is considered to be the driving force behind adaptive responses to exercise [81], it is not a surrogate marker of exercise performance. Therefore, future studies are needed to include another performance-related measurements and training protocols along with soy and whey protein supplementation on individuals to clarify the efficacy of these protein types on exercise performance. A randomized trial on untrained participants administered either leucine-matched soy or whey protein supplementation along with 12-week resistance training [27]. The study findings indicated no difference between protein groups were detected regarding lean body mass and strength after the supplementation period. However, researchers did not adjust total daily protein intake, which could affect the outcomes. Future work involving adjusted total protein intake according to the recommendation for athletes/active individuals, including leucine-matched and dose-matched protein administration, and ≥ 12 weeks of training protocols along with the supplementation is required to elucidate the potential efficacy of protein sources on exercise performance.

Two of the studies that determined aerobic capacity by measuring *V*O_2_max yielded contradictory results [9, 53]. A study on junior judoists found better *V*O_2_max after the SP supplementation (0.5 g/kg BW/day) [53], whereas another study on young trained boxers and road cyclists indicated no alteration in *V*O_2_max after the soy supplementation (42.2 g SP/day) compared to the placebo group [9]. These different results may be due to different study characteristics such as different sports type, age group, exercise protocol applied, amount of SP applied and time at which SP was consumed. The study providing positive findings regarding *V*O_2_max interpreted this result as the high arginine content of SP, which may improve maximal cardiac output by supporting nitrogen oxide (NO) production [53]. Further studies are needed to elucidate this interpretation.

All study results on SP and exercise performance are promising; however, they have some limitations to consider. Although half of the studies monitored the diet during the supplementation period [49, 51, 53], one of them did not give any details about the diet composition [51] and the other one did not include the supplemented protein addition [53]. Only one of them set the protein intake at 1.5 g/kg BW/day for both whey and soy protein groups, but they applied 0.8 g/kg BW/day protein to the placebo groups [49]. Since a meta-analysis [54] and a comprehensive review [76] on protein, muscle protein synthesis and strength have indicated that ~ 1.6 g/kg protein intake is recommended for athletes, positive results with whey and soy protein may be due to the adequate protein intake of protein athletes. Therefore, future studies focussing on adequate total protein intake (~ 1.6 g/kg protein) of all participants, regardless of the protein or placebo group, are required to explain these results. Another limitation is that four of the six studies did not evaluate previous ergogenic aid use prior to the study and one of them included participants who did not use any ergogenic aids at least 1month before the study. However, it is recommended to restrict from ergogenic aids 3 months prior to the study to counteract the possible effects of the supplements [82]. None of them controlled for previous dietary practices such as consuming a vegan, omnivorous, or ketogenic diet, which may be of importance for exercise performance [83–85]. Five of the six studies did not restrict participants from physical activity and alcohol for 48 h prior to the study exercise protocol [9, 47, 51–53], which may be particularly important in the acute exercise studies. Four of the six studies did not mention that the participants were free of injury prior to study [47, 51–53]. As these studies have several limitations, the effectiveness of SP for improving exercise performance has not been fully elucidated.

With its high antioxidant and (poly)phenolic content, high protein quality and fast digestibility, SP is recognised as a promising plant-based protein compared with animal-based proteins in supporting muscle protein synthesis and exercise performance [86, 87]. However, as studies on this interaction have several limitations mentioned above, and future studies that address these limitations are needed to clearly demonstrate the efficacy of SP. In addition, since all studies on athletes were conducted with men only, future studies are required to include both sexes. Furthermore, as suggested by Joanisse et al. (2021) [76], the incorporation of stable isotope techniques as well as omics technologies could greatly contribute to determining the efficacy of SP on exercise performance.

### Clinical and Economic Implications

The present review’s findings have notable clinical implications. Observational research has consistently correlated soy intake with lower risk for chronic diseases. For example, systematic review and dose–response meta-analysis of prospective cohort studies reported an inverse association of the consumption of soy/soy products with cancer and cardiovascular disease mortality [88]. The highest category of soy isoflavone intake yielded a 10% lower risk of all-cause mortality compared with the lowest category of intake. Furthermore, each 5 g/day increase in SP intake was associated with a 12% reduction in breast cancer mortality. The latter finding has important ramifications, since female breast cancer is estimated to be the most commonly diagnosed type of cancer worldwide [89]. The protective capacity of SP against chronic diseases is due to bioactive substances within its matrix of compounds. This includes isoflavones, which have exhibited anti-inflammatory, antineoplastic, antiaggregatory, and antioxidant effects [61]. The latter is particularly relevant to the context of clinical effects in exercising populations. Oxidative stress is considered to be a primary contributing factor to the development plaque that manifests as arteriosclerosis [90]. Oxidative stress is caused by excessive accumulation of reactive oxygen and nitrogen species, resulting in cellular damage. Lipid peroxidation (a biomarker of oxidative stress) was lowered by chronic supplementation of SP (40 g/day) in both men [48] and women [91] undergoing resistance training. Antioxidant effects of SP were superior to whey in both studies.

In addition to reduced oxidative stress, improved serum lipid profile may contribute to SP’s cardioprotective effects. Illustrating this is a meta-analysis by Barańska et al. (2021) [92] involving 23 randomised controlled trials on post-menopausal women. SP significantly decreased low-density lipoprotein (LDL) cholesterol, increased (high-density lipoprotein) HDL cholesterol, and lowered total cholesterol in post-menopausal women. These results echoed a previous meta-analysis by Zhan et al. (2005) [93], who reported that SP resulted in significantly decreased total cholesterol, LDL cholesterol and triacylglycerol in both sexes, with these effects being more pronounced in men than women. It is noteworthy that isolated isoflavone extracts—unlike soy protein containing isoflavones—did not significantly lower total cholesterol.

Common concerns with SP consumption are sexual dysfunction, feminisation and hypogonadism due to the estrogenic potential of isoflavones. However, the evidence for this is limited to single-subject case studies involving the consumption of extraordinarily large doses of isoflavones (~ 360 to 400 mg/day) [94, 95]. Isolated SP contains 0.6–1.0 mg/g protein [96], so achieving these intake levels with SP supplementation alone is very far-fetched. Furthermore, a 41-study meta-analysis by Reed et al*.* (2021) [97] reported no significant effect of SP, soy foods or isoflavone extracts on any of the hormonal parameters tested (total testosterone, free testosterone, estradiol, estrone and sex hormone binding globulin). Importantly, a sub-analysis found no differential influence of higher (> 75 mg) versus lower (< 75 mg) isoflavone doses.

On a final note, economic implications of SP supplementation are intriguing since they present a potential win–win scenario in terms of lower financial cost in addition to the cardiometabolic benefits previously discussed. SP isolate costs approximately 30% less than whey protein on mass-equated basis. Regular consumers of robust doses of whey protein may consider partial replacement with SP for these reasons in addition to a similar [98] or greater [42, 45] muscle anabolic response elicited by a soy–dairy blend compared with whey protein alone. While whey has a greater branched chain amino acid content (and a generally greater essential amino acid content), SP has a substantially greater phenylalanine, arginine, and glycine content than whey protein [41]. It is tempting to speculate that a better mutual amino acid profile and the interaction of non-amino acid constituents underpin the tendency toward an equivalent or greater acute anabolic response from the blend of these proteins.

## Conclusions and Future Perspectives

This systematic review reveals that SPs may increase lean mass during resistance training similarly to whey protein; nevertheless, certain studies indicate that milk and whey proteins may be preferable for increasing lean mass at a faster rate. When compared with whey protein, maltodextrin or placebo, the evidence suggests that long-term SP supplementation along with exercise training may boost antioxidant defense and limit lipid peroxidation. In terms of its effects on hormonal response, there are often contradictory findings regarding the effect of SP supplementation on testosterone and cortisol levels, and on biomarkers associated with muscle androgenic or estrogenic signalling. While soy protein supplementation in athletes/active individuals is promising, the studies included in the review have several limitations, such as heterogeneity of interventions, exercises performed, exercise intensities and frequency and timing of protein intake. Therefore, future studies adjusting total daily protein intake as ~ 1.6 g/protein, including both leucine-matched and dose-matched protein administration, and ≥ 12 weeks of training protocols along with the supplementation are needed. Finally, more research is needed to elucidate the body compositional and exercise performance effects of SPs compared to other proteins in different states of energy balance (i.e. sustained hypo- and hypercaloric conditions).

### Supplementary Information

Below is the link to the electronic supplementary material.

Supplementary file1 (PDF 1118 KB)
